# The Expression, Regulation, and Biomarker Potential of Glypican-1 in Cancer

**DOI:** 10.3389/fonc.2019.00614

**Published:** 2019-07-12

**Authors:** Sen Wang, Yudong Qiu, Bing Bai

**Affiliations:** ^1^Department of Clinical Laboratory Medicine, Nanjing Drum Tower Hospital, Medical School of Nanjing University, Nanjing, China; ^2^Department of Hepatopancreatobiliary Surgery, Nanjing Drum Tower Hospital, Medical School of Nanjing University, Nanjing, China

**Keywords:** heparan sulfate proteoglycan, glypican, GPC-1, cancer, biomarker

## Abstract

Glypican-1 (GPC-1) and other glypicans are a family of heparan sulfate proteoglycans. These proteins are highly expressed on the cell membrane and in the extracellular matrix, functioning mainly as modulators of growth factor signaling. Some of them are abnormally expressed in cancer, possibly involved in tumorigenesis, and detectable in blood as potential clinical biomarkers. GPC-1 is another glypican member that has been found to be associated with some cancers, and has increasingly interested the cancer field. Here we provide a brief review about GPC-1 in its expression, signaling and potential as a cancer biomarker.

## Introduction

As a second leading cause of human death, cancer still remains a major health problem in the world (1). Research has revealed major oncogenic signaling pathways, including cell cycle, histone modification, apoptosis, and other biological processes and cellular pathways (2, 3). Although these are essentially important in understanding of the cancer development, most of these pathway components locate intracellularly, making them neither efficiently accessible therapeutic targets, nor ideal for clinical biomarker discovery.

The roles of the extracellular cues have been increasingly recognized in cancer development, in which they can significantly modulate the hallmarks of cancer (4–6). Heparan sulfate proteoglycans (HSPGs) which are mainly at the cell surface and in the extracellular matrix, have gained considerable scientific interest (7–9). They become a new research topic in the cancer field (10, 11).

Glypicans are one of the HSPG families. These membrane-bound proteins participate in organ development by modulating extracellular growth signals and morphogen gradient formation, and are involved in human overgrowth and skeletal dysplasia problems (12). In some cancers, they are highly expressed, associated with tumorigenesis, and regulating angiogenesis for cancer progression and invasion (13, 14). Their causative role in tumorigenesis is supported by genetic evidence (15).

Like other glypicans, Glypican-1 (GPC-1) is recently found to be overexpressed in certain cancers, and involved in the tumorigenesis of certain cancers (16, 17). Importantly, some studies reported its level was increased in the peripheral blood of patients, holding a great promise as a new glypican biomarker in the cancer field (8, 18).

## Heparan Sulfate Proteoglycans, Glypicans, and GPC-1

The HSPGs are glycosylated proteins composed of a core protein with one or more covalently attached glycosaminoglycan (GAG) chains. GAGs are linear tandem repeats of disaccharide units that consists of an amino sugar (N-acetylglucosamine or N-acetylgalactosamine) together with an uronic sugar (glucuronic acid or iduronic acid) or a galactose. Currently, six GAGs have been found: heparin (HP) and heparan sulfate (HS), chondroitin sulfate (CS), dermatan sulfate (DS), keratan sulfate (KS), and hyaluronic acid (HA), with different amino and uronic sugars in their disaccharide units (19). Except HP and HA that are secreted in free forms without covalent attachment to any proteins, the other four GAGs are bound to a core protein at the Ser residue of a Ser-Gly dipeptide sequence to form a proteoglycan (20). HSPGs are widely present on cell membranes and in extracellular matrices, depending on the structure and the tissue expression of their core proteins. HSPGs are usually divided into three major classes: the glycerophosphatidylinositide (GPI)-anchored type which is at the surface of the membrane (such as glypicans), the transmembrane type (such as syndecans), and the extracellular matrix type (such as agrin and perlecan) (21). HSPGs act as co-receptors for signal transduction, playing important roles in cell growth, immune response, and tumorigenesis, etc. (10, 22, 23).

Glypicans are one of the HSPG families, including glypican-1 (GPC-1) through−6 (GPC-6) in mammals with the main difference in the number of the HS chains and the protein attaching site. These proteins are located on the cell membrane, anchored by glycosylphosphatidylinositol (GPI) which is cleavable by the lipase Notum (24). Glypicans are crucial for cancer cell growth, metastasis, and angiogenesis of many human cancer cell types (13, 15). Abnormal expression of glypicans has been noted in multiple types of cancer. For examples, GPC-3 is closely related to the occurrence and development of tumors, such as human hepatocellular carcinoma, ovarian cancer and melanoma (25–27). GPC-2 is associated with neuroblastoma (28, 29).

GPC-1 is composed of a protein (558 amino acids) with the attachment of three HS chains at S486, S488, and S490, respectively. It has both a membrane-anchored form (by GPI at S530) and a secreted soluble form (30). It can also be cleaved by Notum (14, 31). GPC-1 is mainly expressed in the central nervous system and the skeletal system during embryonic development, and is expressed in most tissues in adults (32). Like other HSPGs and glypicans, GPC-1 functions through binding of growth factors, cytokines, enzymes, viral proteins, and other factors by its HS side chains. It is involved in neurodegeneration and cancer development (33–36).

## GPC-1 Expression in Cancer

Studies have shown that GPC-1 is abnormally expressed in a variety of tumor tissues and is associated with the cancer development. Earlier studies employed northern blot and immunohistochemistry, and found both GPC-1 mRNA and protein expression levels were elevated in the pancreas with cancer, compared to normal controls and the pancreas with chronic pancreatitis (37). This was further confirmed by Kayed et al. who used quantitative PCR, and GPC-1 was demonstrated to be mainly localized in pancreatic cancer cells and adjacent fibroblasts (38). Moreover, the GPC-1 expression was significantly correlated with pathologic grades and clinical stages of the pancreatic cancer, and closely associated with the poor prognosis of patients (39).

Increased expression of GPC-1, but not of other glypicans, was also detected in cultured pancreatic cancer cell lines (16). In this study, knockdown of GPC-1 expression in cells inhibited the mitotic response to fibroblast growth factor−2 (FGF-2), suggesting that GPC-1 might play an important role in the initiation and progression of pancreatic cancer.

GPC-1 expression was also increased in breast cancer tissues (17), ovarian malignant tumors (40), prostate cancerous epithelial cells (41). Moreover, 98.8% of esophageal cancer tissues demonstrated an overexpression of GPC-1 and its association with a poor prognosis (42). However, the expression of GPC-1 in colorectal cancer was controversial. Fernández-Vega et al. reported that both GPC-1 mRNA and protein expression levels were increased in colorectal cancer (43), while De Robertis et al. found the GPC-1 mRNA was decreased in metastatic colorectal cancer and non-metastasis colorectal cancer tissues (44).

Possible mechanisms of GPC-1 expression in cancer might involve microRNA expression and DNA hypomethylation. Normally, microRNA-96-5p and microRNA-149 bind to the 3′-UTR region of GPC-1 transcript to suppress its expression. However, the expression of these two microRNAs is often reduced in the pancreatic cancer (45). In addition, two important regulatory molecules, KRAS and ecotropic viral integration Site 1 (EVI1), are two known drivers of the pancreatic carcinogenesis. They both can upregulate GPC-1 expression, in which EVI1 suppresses the microRNA-96 expression (46). Another important mechanism is about the promoter hypomethylation occurring in the GPC-1 gene in the pancreatic ductal adenocarcinoma, in which the GPC-1 mRNA and protein levels are found to be significantly increased (16).

## GPC-1 Signaling in Cancer

Glypicans mediate signaling in cell proliferation, differentiation, and organ development, by interacting with cell membrane receptors via its HS side chains, including Wnt/β-catenin, Hedgehog (Hh), fibroblast growth factor (FGF), insulin-like growth factor (IGF), vascular endothelial growth factor (VEGF), and transforming growth factor-β (TGF-β), etc (13, 15). The mode of action of GPC-1 is well exemplified in the FGF-2 signaling pathway. By binding to the HS chains of GPC-1, the FGF-2 and its receptor FGFR are more efficiently assembled and stabilized, and the ligand FGF-2 is protected from degradation. Besides, the participation of GPC-1 in the assembly also facilitates the dimerization of the FGFR, leading to the accelerated self-phosphorylation that initiates the signal transduction in protein kinase B (PKB), mitogen-activated protein kinase (MAPK) and other cellular signaling pathways (47, 48).

The altered cellular activities and biological processes induced by GPC-1 might be through the modulation of the FGF-2 signaling, the well-known pathway in the regulation of cell growth, survival, differentiation, and neovascularization, tumorigenesis (49, 50). Qiao et al. showed that GPC-1 expression enhanced the growth of brain endothelial cells and sensitized them to mitogenesis induced by FGF-2. Overexpression of glypican-1 resulted in increased angiogenesis and radiation resistance in brain gliomas (51). Interestingly, GPC-1 increased the level of microRNA-149 through activation of FGFR1, and this microRNA in turn repressed other FGFR1 downstream regulations. This negative feedback loop decreased the endothelial cell response to the angiogenic stimulus of FGF (52). Although GPC-1 is positively involved in the FGFR signaling, this effect might be counteracted by its soluble form secreted in the extracellular space (23).

GPC-1 not only regulates FGF-2, but also modulates the VEGF-A signaling. VEGF is a key factor for angiogenesis, one of the essential biological processes for tumorigenesis (53). Both VEGF-A and FGF-2 are a type of heparin binding growth factors (HBGFs) whose signaling strength and duration might be tuned by GPC-1 (54). Both of their signaling were inhibited after GPC-1 was knocked down in a mouse model of pancreatic cancer (55). Moreover, hepatic endothelial cells isolated from mice lacking GPC-1 demonstrated an attenuated mitogenic response to VEGF-A (56).

**Figure 1:**
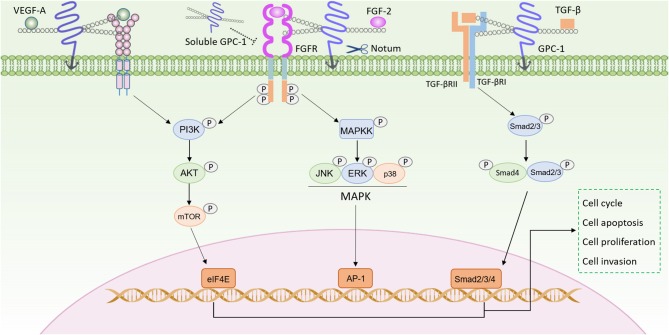
GPC-1 modulates signaling pathways in cancer progression. The HS side chains of GPC-1 bind both growth factors (such as VEGF-A, FGF-2, and TGF-β) and their receptors, to facilitate their assembly for enhanced signaling in PI3K/AKT, MAPK, Smad pathways. GPC-1 can be cleaved by Notum and then released into the extracellular space, which can compete with the GPC-1 anchored on the cell membrane to inhibit its function.

In addition, the GPC-1 also modulates the TGF-β signaling pathway (Figure 1). TGF-β signaling pathway is involved in tumor initiation and progression by regulating cell proliferation, angiogenesis, cancer cell stemness, epithelial mesenchymal transition, invasion and inflammation (57). Here GPC-1 also interacts with the ligand and the receptor to promote the TGF-β signaling. Reduced GPC-1 expression attenuated the TGF-β1 induced inhibition of cell growth, with suppressed Smad2 phosphorylation, and plasminogen activator inhibitor-1(PAI-1) promoter activity in pancreatic cancer cells (58). Kayed et al. analyzed more thoroughly the role of GPC-1 in the TGF-β signaling, in which they found GPC-1 reduction led to a shifted response toward TGF-β, activin-A and the bone morphogenetic protein-2 (BMP-2) upon p21 induction and Smad2 phosphorylation, resulting in inhibited pancreatic cancer cell growth (38).

## GPC-1 as a Clinical Biomarker for Cancer

As the GPC-1 anchored on cell membrane is cleavable and it also has a secreted soluble form, it is detectable in the peripheral blood system, prompting extensive studies carried out on its potential as a clinical biomarker. In 2015, from the 48 proteins identified in the exosomes derived from the cancerous tissue by mass spectrometry and bioinformatics analysis, Melo et al. first reported that GPC-1 could be used as a marker of pancreatic cancer. Subsequently, detection of GPC-1 in human serum exosomes was reported. In breast cancer patients, 75% had higher GPC-1^+^ exosomes than the healthy controls. In pancreatic ductal adenocarcinoma (PDAC), all 190 patient serum samples had higher GPC-1^+^ exosomes than healthy individuals, exhibiting a nearly perfect diagnostic value (~100 and ~100% in the receiver operating characteristic curve). By the Cox multivariate regression analysis, this study also reported the serum GPC-1 exosomes was an independent prognostic marker for disease-specific survival (59).

There are also other reports that employed various methodologies to evaluate the diagnostic potential of GPC-1 in cancers. Qian et al. isolated the serum extracellular vesicles (EVs) and found that the GPC-1^+^ EVs was significantly higher in patients with advanced pancreatic cancer than those in healthy controls (60). Lewis et al. developed an affinity capture elution immunoassay to detect the exosomal GPC-1, which distinguished 20 PDAC patient samples from 11 healthy subjects, with 99% sensitivity and 82% specificity (18). Yang et al. used an advanced multiplexed plasmonic assay and identified a signature of GPC-1 and other four markers for PDAC detection, in which the diagnostic sensitivity and specificity of GPC-1 alone reached 82 and 52%, respectively (61).

The biomarker potential of circulating GPC-1 was also studied in other cancers. The percentage of plasma GPC-1^+^ exosomes significantly increased in colorectal cancer patients than those in healthy controls, and reduced after surgical removal (62). In the urinary sediment samples from 125 patients with prostate cancer and a group of healthy individuals, the sensitivity and specificity of GPC-1 achieved 71 and 76%, respectively (63). Levin et al. also measured GPC-1 in plasma and serum samples and found it was significantly increased in prostate cancer patients as compared to the health cohorts (64). Taken together, these reports suggest that GPC-1 might be a useful marker for the diagnosis of prostate cancer. All these studies about the circulating GPC-1 as a clinical cancer biomarker were summarized in Table 1.

**Table 1:** Circulating GPC-1 as a diagnostic and prognostic marker for cancer.

**Study (Reference)**	**Cancer type**	**Country**	**Case #**	**Sample type**	**Sample preparation**	**Detection method**	**Antibody**	**Results**
Melo et al. (59)	Pancreas cancer	USA	190	Serum	Isolation exosomes using ultracentrifugation	Flow cytometry	PIPA528055, Thermo-Scientific	GPC-1^+^ exosomes (from PDAC, BPD patients and healthy individuals) revealed a near perfect classifier with an AUC of 1.0 (95% CI: 0.956 – 1.0) a sensitivity of 100%.
Lai et al. (65)	Pancreas cancer	USA	29	Plasma	Isolation exosomes using ultracentrifugation	LC-MS	-	Exosomal GPC-1 is not diagnostic for PDAC, whereas a group of microRNA in circulating exosomes is superior to exosomal glypican-1 levels for diagnosing pancreatic cancer.
Frampton et al. (66)	Pancreatic cancer	UK	27	Plasma	Isolation exosomes using ultracentrifugation	ELISA	E9038h, 2BScientific Ltd	There was no significant difference in GPC-1 levels between normal pancreas and PDAC tissues
Qian et al. (60)	Pancreatic cancer	China	28	Plasma	Isolation EVs using exoRNeasy Serum/Plasma Maxi Kit	Flow cytometry	GPC-1 antibody, GeneTex, Inc	Compared with healthy individuals, the levels of GPC-1^+^ EVs were significantly increased in patients with advanced pancreatic cancer.
Lewis et al. (18)	Pancreatic cancer	USA	20	Whole blood, serum, or plasma	Analysis of the biomarkers glypican-1 and CD63 were then performed directly on the chip.	ACEImmunoassay		Twenty PDAC patient samples could be distinguished from eleven healthy subjects with 99% sensitivity and 82% specificity.
Yang et al. (61)	Pancreatic cancer	USA	46	Plasma	Isolation exosomes using ultracentrifugation	Nanoplasmonic sensors	BAF4519, R&D Systems. PA524972, Thermo Fisher.	GPC-1 alone, had a sensitivity of 82% (CI, 60 to 95%) and a specificity of 52% (CI, 30 to 74%) for PDAC detection.
Li et al. (45)	Colorectal cancer	China	102	Plasma	Isolation exosomes using ExoCapTM Kit	Flow cytometry	Anti-GPC-1 antibody, Santa Cruz.	The percentage of plasma GPC-1^+^ exosome was significantly higher in CRC patients before surgical treatment than that in healthy controls and in CRC patients after surgical therapy.
Campbell et al. (63)	Prostate cancer	Australia	41	Urine	Urine cell sediments	Immunofluorescence assay	Monoclonal antibody, MIL-38	Discriminated between prostate cancer and BPH urine specimens with a sensitivity and specificity of 71% and 76%.
Levin et al. (64)	Prostate cancer	Australia	15	Plasma serum	Plasma and serum sample	Luminex assay	Monoclonal antibody, 3G5	Circulating GPC-1 was reduced in prostate cancer patients vs. non- prostate cancer patients.
Lucien et al. (67)	Pancreatic cancer	USA	93	Plasma	Detecting extracellular vesicles based on calibration beads	Nanoscale flow cytometry	PA5-24972, Thermo Fisher.	GPC-1 was unable to discern pancreatic cancer from BPD
Zhou et al. (68)	Pancreatic cancer	China	156	Serum	Serum sample	ELISA	RayBiotech, ELH-GPC-1	The serum GPC-1 cannot be used as a serum diagnostic biomarker for PDAC patients, high levels of serum GPC-1 predict poor prognosis in PDAC patients.

Nevertheless, there are also some studies that yielded controversial results. In a report by Zhou et al. serum GPC-1 level was concluded to be a prognosis factor but not an ideal marker for the clinical diagnosis of PDAC (68). Similar finding was reported by Frampton Prado et al. (66). Lai et al. found the plasma exosomal GPC- 1 level could not differentiate the PDAC patients from the controls, while a panel of microRNAs in the exosomes was a superior pancreatic cancer biomarker instead (65). Moreover, Lucien et al. measured the GPC-1^+^ EVs in the blood samples, and found they were not able to separate the pancreatic cancer patients from those with benign pancreatic disease effectively (67).

There are numerous reasons that might account for these controversial results. First, GPC-1 is not a tissue-specific protein. The human protein atlas database (https://www.proteinatlas.org/) and the comprehensive human tissue proteome analysis that GPC-1 is widely expressed in brain, gastrointestinal tract, urinary, and reproductive systems (69). GPC-1 expressed from the cancerous tissue is probably confounded by these normal secretions from other tissues. Next, in many studies mentioned above, the specificity of the GPC-1 antibody was not seriously validated, which could easily generate false results (70). Many of these antibodies were generated by synthetic short peptides or protein fragments expressed in non-mammalian systems, thus they lacked necessary modifications (especially glycosylations and HS chains on GPC-1) and genuine structures. Ideally, the immunohistochemical staining of GPC-1 should be validated by Western blots with the same antibody, to show whether the blots demonstrated any other non-specific bands and whether the results in these two methods were well correlated. In addition, few studies had thoroughly examined the relationship between the serum GPC-1 levels and the cancer tissue size, the percentage of GPC-1^+^ cells, and the GPC-1 concentration of the total cancer tissue homogenate. In addition, the release of GPC-1 relies on the protease Notum which might not always be expressed in normal amount and activity in cancerous tissues. Notably, many studies used serum as the sample for GPC-1 measurement. The serum differs from the plasma not just in the missing of fibrinogen and other components, but importantly, contains a tremendous amount of active clotting factors, each of which is a highly active protease. It is not known whether any of them might cleave GPC-1, leading to false results. Some studies used the EVs of particular sizes from the plasma for the analysis. However, it remains questionable whether these EVs represent the entire EVs in GPC-1 expression unbiasedly. Besides, the EV extraction for GPC-1 measurements has neither yet proven to be necessary, nor feasible in clinical laboratories. Therefore, more thorough and stringent studies are expected to establish whether GPC-1 in the blood can be a clinical biomarker for certain cancers.

## Concluding Remarks

Glypicans and other HSPGs are very important in the modulation of growth factor signaling. They expressed abnormally in some cancerous tissues, and causatively involved in tumorigenesis. GPC-1 is a new glypican member that has extensively been demonstrated to be increased in certain cancers. Despite a few controversial results, the biomarker potential of GPC-1 deserves further investigation. As membrane and extracellular proteins are more therapeutically accessible and bear more potential to be clinical biomarkers, GPC-1 and other HSPGs will continue to interest the research field for better elucidation of their mechanistic roles and diagnostic values in clinical settings.

## Author Contributions

All authors listed have made a substantial, direct and intellectual contribution to the work, and approved it for publication.

### Conflict of Interest Statement

The authors declare that the research was conducted in the absence of any commercial or financial relationships that could be construed as a potential conflict of interest.
